# Alternative Polyadenylation of Tumor Suppressor Genes in Small Intestinal Neuroendocrine Tumors

**DOI:** 10.3389/fendo.2014.00046

**Published:** 2014-04-15

**Authors:** Anders Rehfeld, Mireya Plass, Kristina Døssing, Ulrich Knigge, Andreas Kjær, Anders Krogh, Lennart Friis-Hansen

**Affiliations:** ^1^Center for Genomic Medicine, Rigshospitalet, University of Copenhagen, Copenhagen, Denmark; ^2^Department of Biology, The Bioinformatics Centre, University of Copenhagen, Copenhagen, Denmark; ^3^Department of Surgical Gastroenterology and Endocrinology, Rigshospitalet, University of Copenhagen, Copenhagen, Denmark; ^4^Department of Clinical Physiology, Nuclear Medicine and PET, Rigshospitalet, University of Copenhagen, Copenhagen, Denmark

**Keywords:** carcinoids, neuroendocrine tumors, gene transcription, molecular biology, polyadenylation

## Abstract

The tumorigenesis of small intestinal neuroendocrine tumors (SI-NETs) is poorly understood. Recent studies have associated alternative polyadenylation (APA) with proliferation, cell transformation, and cancer. Polyadenylation is the process in which the pre-messenger RNA is cleaved at a polyA site and a polyA tail is added. Genes with two or more polyA sites can undergo APA. This produces two or more distinct mRNA isoforms with different 3′ untranslated regions. Additionally, APA can also produce mRNAs containing different 3′-terminal coding regions. Therefore, APA alters both the repertoire and the expression level of proteins. Here, we used high-throughput sequencing data to map polyA sites and characterize polyadenylation genome-wide in three SI-NETs and a reference sample. In the tumors, 16 genes showed significant changes of APA pattern, which lead to either the 3′ truncation of mRNA coding regions or 3′ untranslated regions. Among these, 11 genes had been previously associated with cancer, with 4 genes being known tumor suppressors: *DCC, PDZD2, MAGI1*, and *DACT2*. We validated the APA in three out of three cases with quantitative real-time-PCR. Our findings suggest that changes of APA pattern in these 16 genes could be involved in the tumorigenesis of SI-NETs. Furthermore, they also point to APA as a new target for both diagnostic and treatment of SI-NETs. The identified genes with APA specific to the SI-NETs could be further tested as diagnostic markers and drug targets for disease prevention and treatment.

## Introduction

Small intestinal neuroendocrine tumors (SI-NETs) are a homogeneous group of tumors originating primarily from the serotonin producing enterochromaffin neuroendocrine cells dispersed as solitary cells between the mucosal cells in the small intestine (1). Most SI-NETs tend to be slow growing with a low Ki-67 proliferation index (2). The primary tumor, regional node metastases, and related mesenteric fibrosis may give rise to local symptoms as bowel obstruction and/or vascular encasement. The presence of liver metastases may give rise to the carcinoid syndrome including diarrhea, facial flushing, and on sight cardiac and pulmonary disease, caused by release of serotonin and vasoactive peptides from the liver metastases (2).

Both the incidence and prevalence of SI-NETs have been increasing during the last three decades (3). Nowadays, SI-NETs are the second most prevalent gastrointestinal cancer after colonic cancer (4). Radical surgical intervention is the only curative treatment. However, SI-NETs are difficult to diagnose, as the symptoms often are unspecific. Therefore, many patients are first diagnosed once metastases have occurred and curative surgery is no longer an option (1).

**Figure 1:**
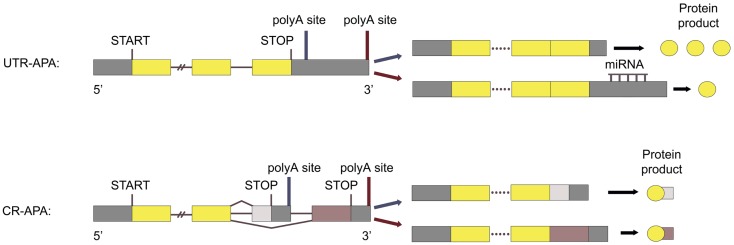
**Types of APA**. *UTR-APA:* APA utilizing polyA sites located in the 3′ UTR of the last exon is called UTR-APA and results in mRNAs with the same coding region, but with different 3′ UTR length. *CR-APA:* APA utilizing polyA sites located in introns, or in the coding region of exons, is called CR-APA and results in mRNAs with 3′ truncated coding regions. Adapted from Ref. (53)

Polyadenylation is a co-transcriptional process that consists of cleavage of the pre-messenger RNA (pre-mRNA) at a polyA site and addition of a polyA tail to the 5′ cleavage product. This process is necessary for normal mRNA 3′-end formation, transcription termination, and mRNA export from the nucleus (5, 6). Studies have shown that almost all eukaryotic pre-mRNAs and several non-coding transcripts have polyA sites and are polyadenylated. More than two-thirds of human genes have multiple polyA sites and can therefore undergo alternative polyadenylation (APA) (7). This produces two or more distinct mRNA isoforms with different 3′ untranslated regions (3′ UTRs). In some cases, APA also produces mRNAs containing different 3′-terminal coding regions. Therefore, APA alters both the repertoire and the expression level of proteins (Figure 1).

Alterations in polyadenylation can cause various diseases (8, 9) and several studies have linked cellular proliferation, transformation, and cancer progression to APA. Proliferation correlates with enhanced proximal polyA site usage leading to expression of mRNAs with 3′ truncated coding regions (10) and truncated 3′ UTRs (5, 10). Cell transformation is associated with 3′ UTR truncation (11–14) and changed APA pattern (10, 15), where transformed cells express mRNAs with shorter 3′ UTRs compared to non-transformed cells with similar proliferation rate (12). Furthermore, 3′ UTR shortening is linked to poor cancer prognosis (16). The APA pattern also differs according to cancer subtype (11), suggesting that the APA pattern could be used as a biomarker for cancer classification.

Considering the previous evidence, we hypothesized that APA leading to 3′ truncation of mRNAs could participate in the tumorigenesis of SI-NETs. To investigate this hypothesis we used high-throughput sequencing data to map polyA sites and characterize APA in SI-NETs genome-wide. We compared polyA site usage in a SI-NET group of three samples, with polyA site usage in a normal neuroendocrine reference sample, pituitary (PIT), and discovered 16 genes with significant APA specific to the SI-NETs, which lead to either 3′ truncation of mRNA coding regions or 3′ UTRs.

## Materials and Methods

### Human clinical samples and RNA isolation

Surgical specimens from SI-NETs were obtained from three patients undergoing surgery at the Department of Surgical Gastroenterology, Rigshospitalet (Table 1). The study was approved by the regional scientific ethical committee (journal number 01 313726). Signed, informed consent was obtained from the patients. Immediately after tumor resection, tumor tissue samples from the surgical specimens were placed in RNAlater^®^ (Applied Biosystems, Carlsbad, CA, USA) for overnight incubation. Samples were subsequently stored at −80°C until RNA was extracted using TRIzol (Life Technologies, Carlsbad, CA, USA). The reference sample (PIT), human pituitary RNA, was purchased from BioChain (Hayward, CA, USA).

**Table 1:** **SI-NET biopsies**.

Identifier	Tissue of origin	Sex/age	Synaptophysin	Chromogranin	Serotonin	CD117
NE MTT	Ileum	M/73	+	+	+	−
NE CT1	Ileum	F/66	+	+	NA	NA
NE 2TC	Ileum	M/73	+	+	+	−

### Sequencing

Total RNA from the three SI-NETs and the reference sample (PIT) were sequenced with single-molecule direct RNA-sequencing (DRS) at Helicos BioSciences (Boston, MA, USA) (17). The 3′ blocking reaction was performed with 3′ deoxyATP (Jena Biosciences, Germany) by incubating the reaction mixture at 37°C for 30 min.

### Read processing and mapping

Raw DRS reads starting with one or more Ts were trimmed as these can be due to inefficient locking of the RNA with the polyT primer at the beginning of the sequencing (17). Trimmed reads were mapped to the genome (hg19) with HeliSphere software (http://sourceforge.net/projects/openhelisphere/) using a seed length of 18 nt and allowing only one mismatch in the seed region. Only uniquely mapped reads with a minimum length of 21 nt and a minimum score of 4 where further analyzed. We additionally discarded all reads that may arise from internal priming events. DRS reads are sequenced by synthesis, and thus, they are reverse complementary to the original RNA fragments (17). We checked the 10-nt immediately downstream of the read end in the complementary strand for adenines. In the case that the first six or more of these nucleotides were adenines the read was discarded (14). A summary of the data after the different steps can be seen in Table 2.

**Table 2:** **Read processing statistics**.

	NE MTT	NE CT1	NE 2TC	PIT
Raw reads	4895411	10878794	8859412	9773879
Uniquely mapped reads (>21 nt)	2012076	4465454	3675490	4027944
Reads filtered after removing internal priming events	1918905	4266418	3499253	3816258
Clusters (including singletons)	686450	1352151	1296130	1289606
PolyA sites	221147	398528	379890	385451

### Identification of polyA sites

**Figure 2:**
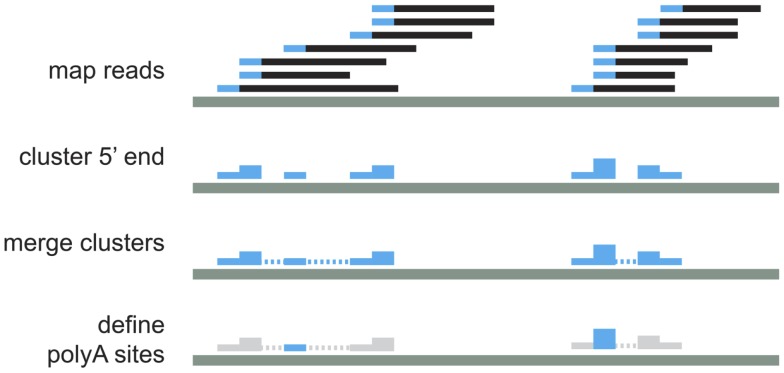
**Schematic representation of read processing and clustering**.

**Figure 3:**
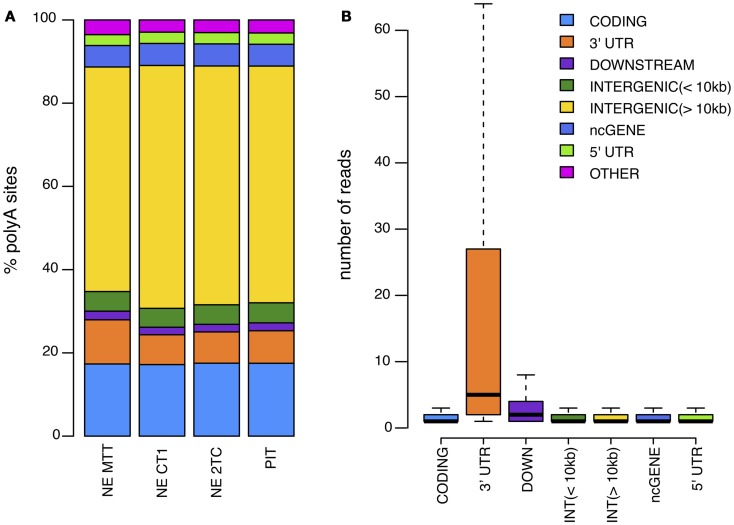
**(A)** Distribution of identified polyA sites across the genome for each of the samples analyzed. **(B)** Average number of reads per polyA site in different genomic regions.

It is known that the cleavage process is inefficient and can vary within a few nucleotides (18). To account for this variability, we cluster iteratively the 5′ end of mapped reads that were <24 nt apart (14, 18). For each of these clusters, the cleavage site is defined as the median position of the cleavage sites in the cluster (Figure 2). As previous works have shown that APA is very common and that even very infrequently used polyA sites are real (7), we kept all polyA sites that have at least one read in at least two samples, or that have two or more reads. Next, each of the clusters was annotated according to the location of the cleavage site using the genome annotation from Ensembl66 (19). PolyA sites were classified as 5′ UTR, CODING, or 3′ UTR if overlapping these regions of coding genes; ncGENE if overlapping a non-coding gene; PROMOTER if they were located in the 1000 bases upstream of a gene; DOWNSTREAM if they were located in the 1000 bases downstream of a gene; and INTERGENIC otherwise (Figure 3A). PolyA sites found in both introns and exons of the coding region are classified as CODING, as usage of these changes the coding region of the mRNA. As DOWNSTREAM polyA sites (≤1 kB) have many more reads than other intergenic polyA sites (Figure 3B), we associated the polyA sites in this proximal intergenic region (≤1 kB) to the upstream gene and included them in the analysis of APA.

### Identification of APA in SI-NETs

We restricted the analysis to protein coding genes. For each gene, we selected only those polyA sites that have at least 10% of all reads mapped to a gene in a given sample. By doing this, we excluded from the analysis those polyA sites that could have little or no impact on the expression levels or the regulation of the mRNA. For each gene we created a contingency table with the counts of reads for each polyA site, where in each column we had represented a polyA site identified in one or more samples and on each row we had each of the samples. To identify differential polyadenylation we performed fisher exact tests with R (R Development Core Team 2008) for each pair of samples. After multiple test correction, only those cases in which no statistical difference was found between the samples in the SI-NET group [false discovery rate (FDR) >0.1] and significant differences were found for all the samples in the SI-NET group compared to the reference sample (PIT) (FDR <0.1) were considered significant.

### Manual selection of APA cases

Firstly, we selected the genuine cases of APA, by manually removing genes if: (1) there is no APA within the gene alone, but only in tandem transcripts containing the gene (11 genes removed). (2) APA is caused by an overlapping gene (one gene removed). Secondly, we selected the cases of APA leading to a reasonable expression in the SI-NETs, by manually removing genes if: (3) the implicated polyA sites have <10 reads in 2/3 of the SI-NETs (11 genes removed). Lastly, we selected the cases of APA, which lead to 3′ truncation of the mRNA, by manually removing genes if: (4) the most distal polyA site of the gene is primarily used in the SI-NET group (14 genes removed). (5) The most proximal of the polyA sites is primarily used in the reference sample (PIT) (eight genes removed). The manually removed genes are listed in Table S2 in Supplementary Material.

### Quantitative real-time-PCR validation

cDNA was synthesized from the RNA samples using High Capacity cDNA Reverse Transcription Kit (Applied Biosystems, Foster City, CA, USA) according to the manufactures instructions. Quantitative real-time-PCR (Q-RT-PCR) was performed with SYBR^®^ GreenER™ qPCR SuperMix for ABI PRISM^®^ (Life Technologies, Carlsbad, CA, USA) on the ABI PRISM^®^ Instrument (Applied Biosystems, Foster City, CA, USA). Primer sequences were as follows:

- *DCC*, upstream of proximal polyA site:
  *DCC-INT5-FW: 5*′*-acccgtcaccttcttttcct-3*′
  *DCC-INT5-RV: 5*′*-caccgtgccctattttgtct-3*′
- DCC, upstream of distal polyA site:
  *DCC-INT13-FW: 5*′*-aaaaacgcagtacggcaact-3*′
  *DCC-INT13-RV: 5*′*-tgggatgggttctttctcag-3*′
- PDZD2, upstream of proximal polyA site:
  *PDZD2-INT3-FW: 5*′*-gtgagaccctgtccctacca-3*′
  *PDZD2-INT3-RV: 5*′*-gtaaagcaaccatgccacct-3*′
- PDZD2, upstream of distal polyA site:
  *PDZD2-EX26-FW: 5*′*-gtcggcctagagaggtcctt-3*′
  *PDZD2-EX26-RV: 5*′*-catgcaccttgcactgactt-3*′
- LRRFIP1, upstream of proximal polyA site:
  *LRRFIP1-INT39-FW: 5*′*-gtcctgcagaaccaaagagc-3*′
  *LRRFIP1-INT39-RV: 5*′*-aagctttcctgacggtctga-3*′
- LRRFIP1, upstream of distal polyA site:
  *LRRFIP1-EX76-FW: 5*′*-cgctggaagtgatgatcaaa-3*′
  *LRRFIP1-EX76-RV: 5*′*-gcttggtaatggcttgtggt-3*′

## Results

### Global characterization of polyA site usage reveals similarity between SI-NETs

Our first objective was to map polyA sites in SI-NETs genome-wide and characterize APA. To perform this characterization, total RNA from three SI-NET samples (NE MTT, NE CT1, and NE 2TC) and from a reference sample (PIT) were sequenced using DRS (17). DRS is a well established method, which have been used in several other studies to map polyA sites genome-wide (14, 17, 20, 21). The SI-NETs originate from neuroendocrine cells of the small intestine, which are solitary interspersed cells (22). It is very difficult to identify and collect enough normal neuroendocrine cells as a control sample, and scrapings of the mucosa adjacent to the tumors are thus normally used as reference samples (23). However, we chose to use pituitary as reference sample as we expected that the neuroendocrine cells of the pituitary are phenotypically closer to the neuroendocrine cells of the small intestine than the mixture of different cell types from the mucosal scrapings. From the DRS we obtained a total of 4895411 (NE MTT), 10878794 (NE CT1), and 8859412 (NE 2TC) reads. The final filtered reads were mapped to 221147 (NE MTT), 398528 (NE CT1), and 379890 (NE 2TC) polyA sites. These sites mapped to various genomic regions in the samples (Figure 3). Fisher exact tests found 11031 polyA sites, located in 97% of the expressed protein coding genes, with no statistical difference in usage between the individual SI-NETs (FDR >0.1). This shows similarity between the three SI-NET samples. The sequencing data obtained by DRS have been deposited to the National Center for Biotechnology Information (NCBI)’s Gene Expression Omnibus (GEO), with accession number: GSE56657.

### Alternative usage of polyA sites in the SI-NETs

The second aim of the project was to identify protein coding genes in SI-NETs with a differential APA pattern compared to the reference sample. Using fisher exact tests we identified 61 genes with statistically significant APA between the SI-NET group and the reference sample (Table S1 in Supplementary Material). From these 61 genes, 45 genes were removed manually, to select only the genuine cases of APA, which lead to either 3′ truncation of mRNA coding regions or 3′ UTRs (Table S2 in Supplementary Material). The 16 selected genes with APA specific to the SI-NETs are described below, including a description of the possible implications for protein isoform and expression (Table S3 and Figure S1 in Supplementary Material). Interestingly, 11 out of these 16 genes have previously been associated with cancer, with 4 of the genes being known tumor suppressors. These 16 genes with APA specific to the SI-NETs could be tested as diagnostic markers and drug targets for disease prevention and treatment.

### Genes with UTR-APA specific to the SI-NETs

Of the 16 genes with APA specific to the SI-NETs, 9 genes had significant UTR-APA leading to 3′ UTR shortening in a larger proportion of mRNAs than in the reference sample. These genes are: *PSMD8, DACT2, TM9SF3, CD59, ANKH, CIAO1, SRSF5, MRSP16*, and *NDUFA6*. The shortening of 3′ UTRs plays an important part in post-transcriptional regulation, as the 3′ UTR contains target sites for microRNAs (miRNAs), which control mRNA turnover and translation rate (24). Because of miRNA target site loss, mRNAs with shorter 3′ UTRs are more stable and produce more protein (5, 11, 12). We mapped the target sites of all human miRNAs to the 3′ UTRs of genes displaying UTR-APA (Figure S2 in Supplementary Material). The miRNA target site predictions were downloaded from microRNA.org (25). Only conserved miRNAs with a good score were mapped. We then compared the miRNA target sites in the 3′ UTRs of the nine genes to miRNA-profiles of five primary SI-NET samples from another study (26). We considered only the miRNAs that were detected in a minimum of four out of these five primary SI-NET samples. For all nine genes, except *DACT2*, the 3′ UTR shortening leads to loss of miRNA target sites for miRNAs detected in SI-NETs, suggesting an upregulated expression for these genes. See Table 3 for a detailed description of the genes with UTR-APA.

**Table 3:** **Genes with UTR-APA specific to the SI-NETs**.

Gene	Function	Role in cancer	APA	Functional consequence
*PSMD8*	Subunit of the proteasome	Overexpressed in breast cancer (27)	Increased usage of a proximal polyA site in the 3′UTR	Shortening of 3′UTR by 325 nt with loss of miRNA target sites for three miRNAs present in SI-NETs: hsa-mir-22*, hsa-mir-192*, and hsa-mir-1263
*DACT2*	Negative regulator of the TGF-β/nodal signaling pathway	Tumor suppressor (28)	Increased usage of two proximal polyA sites in the 3′UTR	Shortening of 3′UTR by up to 12113 nt without loss of miRNA target sites
*TM9SF3*	Transmembrane protein with unknown function	Unknown	Increased usage of two proximal polyA sites in the 3′UTR	Shortening of 3′UTR by up to 2848 nt with loss of miRNA target sites for 13 miRNAs present in SI-NETs: hsa-mir-1826, hsa-mir-338-5p, hsa-mir-552, hsa-mir-532-3p, hsa-mir-7-2*, hsa-mir-7-1*, hsa-mir-10b*, hsa-mir-140-3p, hsa-mir-193b*, hsa-mir-550*, hsa-mir-200c*, hsa-mir-17*, and hsa-mir-93*
*CD59*	Membrane complement regulatory protein	Overexpressed and anti-apoptotic (29)	Increased usage of two proximal polyA sites in the 3′UTR	Shortening of 3′UTR by up to 4707 nt with loss of miRNA target sites for one miRNA present in SI-NETs: hsa-mir-629
*ANKH*	Pyrophosphate transport regulator	Amplified and suggested overexpressed (30)	Increased usage of a proximal polyA site in the 3′UTR	Shortening of 3′UTR by 4879 nt with loss of miRNA target sites for eight miRNAs present in SI-NETs: hsa-mir-330-3p, hsa-mir-29b-2*, hsa-mir-1275, hsa-mir-625, hsa-mir-452, hsa-mir-628-3p, hsa-mir-30a*, and hsa-mir-30e*
*CIAO1*	Role in iron–sulfur protein biogenesis	Interacts with tumor suppressor WT1 (31)	Increased usage of two proximal polyA sites in the 3′UTR	Shortening of 3′UTR by up to 2581 nt with loss of miRNA target sites for nine miRNAs present in SI-NETs: hsa-mir-550*, hsa-mir-200c*, hsa-mir-214*, hsa-mir-423-5p, hsa-mir-660, hsa-mir-29b-1*, hsa-mir-1246, hsa-mir-7-1*, and hsa-mir-7-2*
*SRSF5*	Splice factor	Overexpressed in breast cancer and anti-apoptotic (32)	Increased usage of a proximal polyA site in the 3′UTR	Shortening of 3′UTR by 312 nt with loss of miRNA target sites for seven miRNAs present in SI-NETs: hsa-mir-338-5p, hsa-mir-628-3p, hsa-mir-409-3p, hsa-mir-183*, hsa-mir-330-3p, hsa-mir-7-1*, and hsa-mir-7-2*
*MRSP16*	Mitochondrial ribosomal protein	Unknown	Increased usage of a proximal polyA site in the 3′UTR	Shortening of 3′UTR by 1888 nt with loss of miRNA target sites for 16 miRNAs present in SI-NETs: hsa-mir-671-5p, hsa-mir-193b*, hsa-mir-551b*, hsa-mir-338-5p, hsa-mir-30b*, hsa-mir-125b-2*, hsa-mir-330-3p, hsa-mir-7-1*, hsa-mir-7-2*, hsa-mir-188-5p, hsa-mir-769-5p, hsa-mir-324-3p, hsa-mir-552, hsa-mir-193a-5p, hsa-mir-501-5p, and hsa-mir-362-5p
*NDUFA6*	NADH dehydrogenase	Unknown	Increased usage of a proximal polyA site in the 3′UTR	Shortening of 3′UTR by 563 nt with loss of miRNA target sites for six miRNAs present in SI-NETs: hsa-mir-552, hsa-mir-21*, hsa-mir-664*, hsa-mir-505*, hsa-mir-550, and hsa-mir-500*

### Genes with CR-APA specific to the SI-NETs

Seven of the genes identified had significant CR-APA leading to 3′ truncated coding regions in a larger proportion of mRNAs than in the reference sample. These genes are: *DCC, PDZD2, MAGI1, ZCWPW2, LRRFIP1, RIC3*, and *THADA*. CR-APA does not lead to nonsense-mediated decay and the expressed mRNAs thus translate into C-terminally truncated protein isoforms that could be non-functional or have functions different from the full-length protein (33). The seven genes with CR-APA are described in detail in Table 4.

**Table 4:** **Genes with CR-APA specific to the SI-NETs**.

Gene	Function	Role in cancer	APA compared	Functional consequence
*DCC*	Receptor for the axon guidance molecule nextrin	Tumor suppressor (34)	Increased usage of a proximal polyA site located in intron 5	Usage of this polyA site leads to expression of an mRNA lacking 24 exons in the 3′end, which translates into a highly C-terminally truncated DCC protein containing only 5 Ig-like domains and lacking both the nextrin binding domain, the transmembrane domain, and the intracellular domain. Thus, it lacks the parts responsible for the tumor suppressive functions (34)
*PDZD2*	PDZ domain-containing protein	Can be cleaved near its C-terminus to generate a secreted form sPDZD2 with tumor suppressive function (35, 36)	Increased usage of two proximal polyA sites located in intron 2	Usage of these polyA sites leads to expression of an mRNA lacking 23 exons in the 3′end, which translates a highly C-terminally truncated PDZD2 protein, lacking the ability to form sPDZD2 and thus the tumor suppressive function (35)
*MAGI1*	Scaffolding protein localized at cell–cell contacts, containing PDZ domains	Tumor suppressive and anti-metastatic, through β-catenin recruitment to the cell membrane and thus inhibition of the Wnt/β-catenin signaling pathway (37)	Increased usage of two proximal intronic polyA sites located in the intron 2	Usage of these polyA sites leads to expression of an mRNA lacking 21 exons in the 3′end, which translates a highly C-terminally truncated MAGI1 protein, lacking all PDZ domains and thus the tumor suppressive function (37)
*ZCWPW2*	Zinc finger protein	Unknown	Increased usage of a proximal polyA site located in intron 5	Usage of this polyA site leads to expression of an mRNA lacking five exons in the 3′end, which translates into a highly C-terminally truncated ZCWPW2 protein
*LRRFIP1*	Transcriptional repressor	Overexpressed in cancer cell lines (38) and pro-metastatic (39)	Increased usage of a proximal polyA site located in the fifth last intron	Usage of this polyA site leads to the expression of an mRNA lacking five exons in the 3′end. The 3′-end of this mRNA isoform corresponds to the 3′-end of the 4.2-kB mRNA overexpressed in cancer cell lines (38)
*RIC3*	Chaperone protein	Unknown	Increased usage of a proximal intronic polyA site located in intron 1	Usage of this polyA site leads to the expression of an mRNA lacking five exons in the 3′end, giving rise to C-terminally truncated RIC3 protein
*THADA*	Unknown	Frequently truncated in thyroid adenomas and the truncated allele is hypothesized to play a role in the thyroid tumorigenesis (40)	Increased usage of a proximal polyA site located in the intron 22	Usage of this polyA site leads to the expression of an mRNA lacking 16 exons in the 3′end, i.e., more than in the thyroid adenomas (40), which translates into highly C-terminally truncated THADA protein

### Q-RT-PCR validation of APA

Three genes were selected for Q-RT-PCR validation: *DCC, PDZD2*, and *LRRFIP1*. cDNA was synthesized from total RNA from all four samples. Primers were designed as sets targeting the regions just upstream of a proximal and a distal polyA site with significant differential usage between the SI-NET group and PIT. The proximal primer sets targeted intronic sequences, corresponding to 3′ UTRs of alternative last exons, and the distal primer sets targeted either intronic sequences, corresponding to 3′ UTRs of alternative last exons, or the 3′ UTRs of the last exon. As the proximal primer sets targeted intronic sequences, the detection of a product from these sets could only come from usage of the adjacent polyA site, as the intron is otherwise spliced out. Proximal and distal isoforms were thus individually measured. The genes were used as their own controls, as there were two sets of primers for each gene; one upstream of a proximal polyA site and one upstream of a distal polyA site. The Ct values of the proximal and distal amplicon were thus compared. Q-RT-PCR was run on triplicates of each sample with all six primer sets. Median Ct values from the triplicates were used for further analysis with individual Ct values deviating more than two from the median considered as outliers and removed (2 of the 72 Ct values removed). In contrast to our results obtained from DRS (Table 5), we found a higher expression of the proximal isoform of *DCC* and *LRRFIP1*, as well as a lower expression of the proximal isoform of *PDZD2* in all three SI-NETs as well as in the reference sample (Table 6). However, when comparing the results for the SI-NETs with our reference sample, we found a higher expression of the proximal *LRRFIP1* isoform in all three SI-NETs compared to our reference sample. For *DCC* and *PDZD2*, we found a higher expression of the proximal isoform in two out of the three SI-NETs compared to our reference sample (Table 7). These Q-RT-PCR results validate the presence of the polyA sites and the APA of these genes. They also indicate that these three genes indeed undergo differential APA leading to the expression of a larger amount of mRNAs with 3′ truncation of the coding region in the SI-NETs than in the reference sample.

**Table 5:** **Expression ratio of (short isoform/long isoform) derived from DRS data**.

	*DCC* proximal/distal	*PDZD2* proximal/distal	*LRRFIP1* proximal/distal
NE 2TC	25	14	3.725490196
NE CT1	156	15.66666667	2.173469388
NE MTT	Inf	36	3.25
PIT	0.128205128	0.652631579	0.735294118

**Table 6:** **Expression ratio of (short isoform/long isoform) derived from Q-RT-PCR data**.

	*DCC* proximal/distal	*PDZD2* proximal/distal	*LRRFIP1* proximal/distal
NE 2TC	3.853506079	0.026305316	25.55396049
NE CT1	2.887782327	0.012957265	14.26667269
NE MTT	1.019851667	0.032658206	30.08928667
PIT	2.030313016	0.025800759	3.645085529

**Table 7:** **Expression ratio of (short isoform/long isoform) normalized to reference sample, derived from Q-RT-PCR data**.

	*DCC*	*PDZD2*	*LRRFIP1*
NE 2TC	1.897986	1.019556	7.010524
NE CT1	1.422334	0.502205	3.913947
NE MTT	0.502313	1.265785	8.254755

## Discussion

This is the first study to map polyA sites and characterize APA genome-wide in SI-NETs. To map the polyA site usage genome-wide we sequenced total RNA from three SI-NET samples (NE MTT, NE CT1, and NE 2TC) and a reference sample (PIT) using DRS (17). DRS is a well established method, which have been used in several other studies to map polyA sites genome-wide (14, 17, 20, 21). Selected polyA sites have been confirmed in all cases with PCR in three of those studies (14, 17, 21). We selected three genes for Q-RT-PCR validation: *DCC, PDZD2*, and *LRRFIP1*. For these three genes we validated the presence of all tested polyA sites with Q-RT-PCR and thus the APA of the selected genes. However, we did not find the same ratio of usage between the proximal and distal polyA sites with Q-RT-PCR as we found with DRS (Tables 5 and 6). When comparing our Q-RT-PCR results in the SI-NETs with the reference sample, they however indicate that the three genes in the SI-NETs indeed undergo differential APA leading to expression of a larger amount of mRNAs with 3′ truncation of the coding region than in the reference sample (Table 7). The quantitative differences found in expression ratios of the short and long isoforms derived from DRS and Q-RT-PCR could be due to biases and artifacts introduced during the synthesis of cDNA, contamination of cDNA samples with genomic DNA and problems with one or more of the primer sets.

When studying SI-NETs the optimal control sample would be normal small intestinal neuroendocrine cells. These solitary cells, which are interspersed among the other cells of the mucosa, are however difficult to isolate (23). In one study, human neuroendocrine cells have been isolated by pronase/collagenase digestion, gradient centrifugation, and FACS, but this method is complicated and requires at least 5–8 cm of normal ileum (41). Additionally, it is not certain if the RNA content is damaged during those processes. Because of these difficulties, scrapings from the mucosa adjacent to the tumors are normally used as reference samples. This mucosa is however composed of a mix of various cell types, primarily enterocytes and goblet cells. We believe that this mixture of cells from the mucosa, despite a common origin with the neuroendocrine cells (42), is phenotypically farther from intestinal neuroendocrine cells than neuroendocrine cells elsewhere in the body. We therefore chose to use pituitary as a reference sample as it mainly contains neuroendocrine cells.

Using Fisher exact tests, we found 11031 polyA sites, located in 97% of the expressed protein coding genes, with no statistical difference in usage between the individual SI-NETs (FDR >0.1). This shows similarity between the three SI-NET samples. Using the same Fisher exact tests to compare APA in the SI-NETs and in the reference sample, we identified 16 genes with significant APA specific to the SI-NETs, which lead to either 3′ truncation of mRNA coding regions or 3′ UTRs (Figure S1 in Supplementary Material). These 16 genes with APA specific to the SI-NETs could be tested as diagnostic markers and drug targets for disease prevention and treatment.

Nine of the 16 genes had significant UTR-APA in the SI-NETs leading to 3′ UTR shortening in a larger proportion of mRNAs than in the reference sample. Interestingly, four genes out of the nine with UTR-APA have been found to be either overexpressed in cancer: *PSMD8* (27), *CD59* (29), and *SRSF5* (32) or is suggested to be overexpressed: *ANKH* (30). The UTR-APA in these genes produces mRNA isoforms that lack one or more target sites for miRNAs present in SI-NETs. These mRNAs consequently escape a part of the miRNA regulation and, therefore, should be more stable and produce more protein (5, 11, 12). Thus, the changes predicted in these nine genes suggest that UTR-APA could contribute to the SI-NET tumorigenesis. One of these genes, *DACT2*, was previously found as a tumor suppressor protein (28). The UTR-APA in this gene leads to 3′ UTR shortening in a larger proportion of mRNAs in the SI-NETs than in the reference sample. However, as no miRNA target sites are found in the shortened part of the 3′ UTR, it is unclear which role this 3′ UTR region has. Apart from miRNAs, RNA binding proteins (RBPs) also target the 3′ UTR of mRNAs and can affect the stability of mRNAs (43). Thus, it is possible that the shortening of the 3′ UTR affects the stability of *DACT2* by removing putative binding sites of RBPs.

Seven of the 16 genes had significant CR-APA in the SI-NETs leading to 3′ truncated coding regions in a larger proportion of mRNAs than in the reference sample. CR-APA alters the coding region of mRNAs and thus the isoform of the produced protein. Remarkably, three out of the seven genes with CR-APA have tumor suppressive functions: *DCC* (34), *PDZD2* (35), and *MAGI1* (37). The CR-APA in these genes produces mRNA isoforms lacking several 3′-terminal exons, translating into highly C-terminally truncated proteins. These protein isoforms lack the parts responsible for the tumor suppressive functions. The CR-APA in *DCC, PDZD2*, and *MAGI1* could thus likely be contributing to the tumorigenesis in SI-NETs. *DCC* is located in chromosome 18, where there have been reported frequent copy-number losses in SI-NETs (44, 45). The copy-number losses might truncate the *DCC* gene, forcing the usage of the proximal polyA site. Another gene with CR-APA, *THADA*, has been hypothesized to contribute to the tumorigenesis of thyroid adenomas in its truncated form (40). The CR-APA found in the SI-NETs produces an mRNA even more 3′-terminally truncated and could thus also be a candidate contributing to the tumorigenesis of SI-NETs. One gene stands out in the CR-APA group, *LRRFIP1*, as it has been shown to be overexpressed in cancer cell lines (38) and to be pro-metastatic (39). The CR-APA of this gene in the SI-NETs gives rise to an mRNA isoform, with a 3′-end corresponding to the 3′-end of the 4.2 kB mRNA overexpressed in cancer cell lines (38). CR-APA of *LRRFIP1* could thus also be a contributor to the tumorigenesis in SI-NETs, as the major mRNA isoform expressed in the SI-NETs is likely equal to that overexpressed in the cancer cell lines.

The suggested participation of APA in the tumorigenesis of SI-NETs could lead to new medical treatments of SI-NETs. This is particularly interesting as polyA sites of interest can be very specifically and effectively blocked and patterns of APA can thus be changed. Different techniques utilizing antisense elements have already been used to effectively inhibit polyA site usage. They include antisense oligonucleotides (46), siRNAs (47), and U1 modifications (48–52). Such techniques could, e.g., be used to force the expression of the full-length mRNAs of the tumor suppressor genes *DCC, PDZD2*, and *MAGI1*.

As this study was performed on only three SI-NET samples the findings should be confirmed in a larger cohort. Preferably, future studies on APA in SI-NETs should also include alternative reference tissues and use different methods for characterizing poly(A) site usage. The findings could also be tested in other types of NETs. If the findings are confirmed, the effects of blocking the implicated proximal poly(A) sites with antisense elements should be tested experimentally.

In conclusion, we have mapped for the first time polyA sites genome-wide in SI-NETs. We found 11031 polyA sites, located in 97% of the expressed protein coding genes, with no statistical difference in usage between the individual SI-NETs. This shows similarity between the three SI-NET samples. In the SI-NETs, we identified 16 genes with significant APA leading to either 3′ truncation of coding regions or 3′ UTRs in a larger proportion of mRNAs than in the reference sample (Tables 3 and 4). The APA was confirmed in three genes using Q-RT-PCR: *DCC, PDZD2*, and *LRRFIP1*. However, we did not find the same expression ratios between the proximal and distal isoforms for these genes with Q-RT-PCR as with DRS (Tables 5 and 6).

As 11 of the 16 identified genes have previously been associated with cancer, it is likely that APA in these genes could take part in the SI-NET tumorigenesis. Future studies will have to confirm these findings in a larger cohort. The identified 16 genes with APA specific to the SI-NETs could be tested as diagnostic markers and as drug targets for disease prevention and treatment.

## Author Contributions

Study concept and design (Anders Rehfeld, Lennart Friis-Hansen). Acquisition of data (Anders Rehfeld, Lennart Friis-Hansen). Analysis and interpretation of data (Anders Rehfeld, Mireya Plass, Anders Krogh, Lennart Friis-Hansen). Drafting of the manuscript (Anders Rehfeld, Mireya Plass, Lennart Friis-Hansen). Critical revision of the manuscript for important intellectual content (Anders Rehfeld, Mireya Plass, Kristina Døssing, Ulrich Knigge, Andreas Kjær, Anders Krogh, Lennart Friis-Hansen). Statistical analysis (Mireya Plass, Anders Krogh). Obtained funding (Anders Rehfeld, Anders Krogh, Lennart Friis-Hansen). Administrative, technical, or material support (Lennart Friis-Hansen). Study supervision (Lennart Friis-Hansen).

## Conflict of Interest Statement

The authors declare that the research was conducted in the absence of any commercial or financial relationships that could be construed as a potential conflict of interest.

## Supplementary Material

The Supplementary Material for this article can be found online at http://www.frontiersin.org/Journal/10.3389/fendo.2014.00046/abstract
