# Supplementary material for: Alternative Polyadenylation of Tumor Suppressor Genes in Small Intestinal Neuroendocrine Tumors
Source: Front Endocrinol (Lausanne). 2014 Apr 15;5:46. doi: 10.3389/fendo.2014.00046 (PMC3995063; doi:10.3389/fendo.2014.00046)
Supplement: Supplementary file 1 [file Data_Sheet1.PDF]

Supplementary figure 1: Bargraph images of polyA site usage in the 16 manually selected genes, generated using the UCSC Genome Browser. The three tracks in red: NE_MTT_minus/plus, NE-CT1_minus/plus and NE_2TC_minus/plus represent the three small intestinal tumor samples. The track in blue: PIT_minus/plus represents the reference pituitary sample. For each track the polyA site usage is represented by the vertical bargraph going from 1 to the maximal value within the given genomic region.

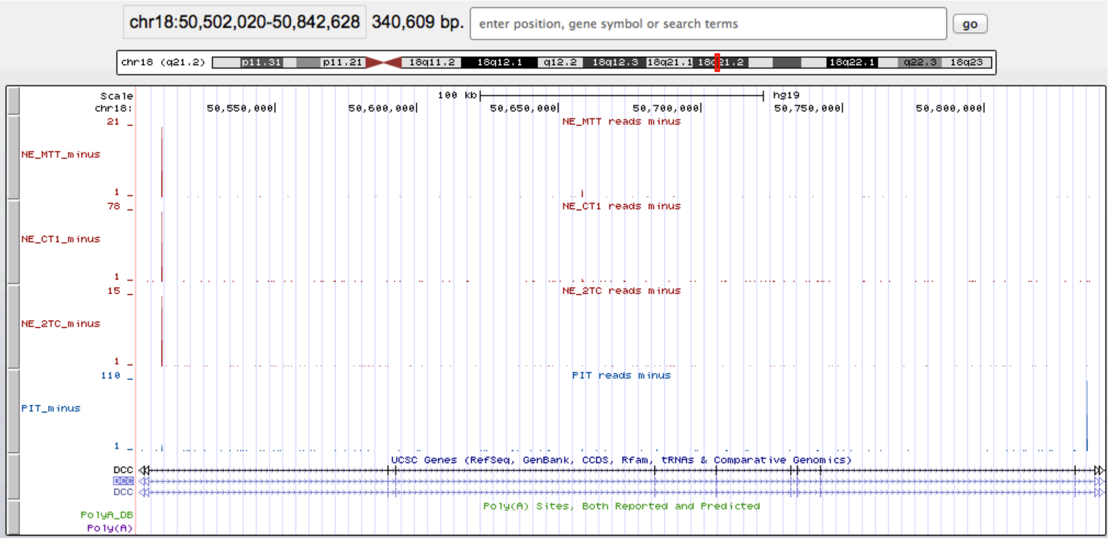

DCC

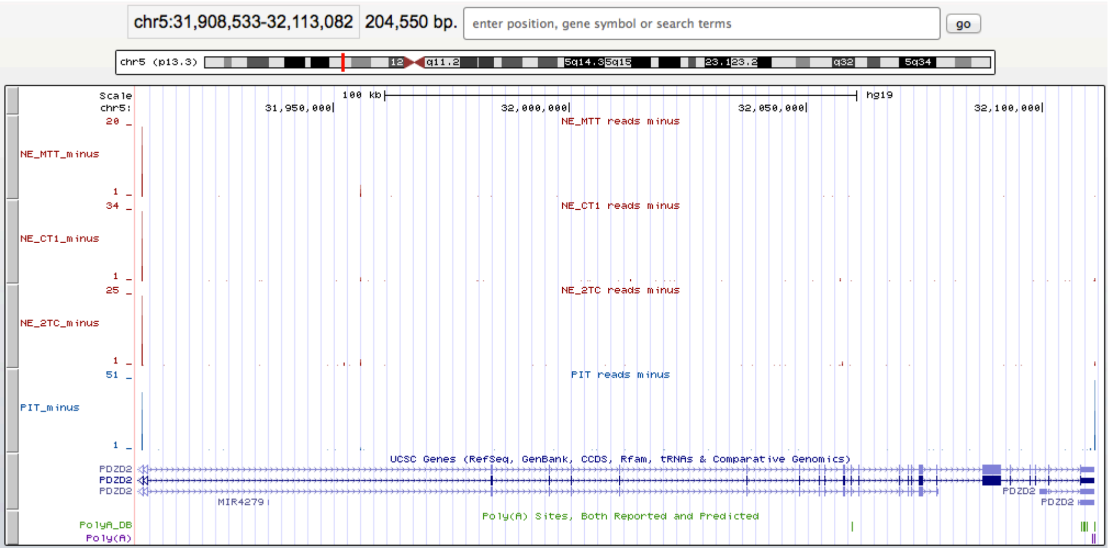

PDZD2

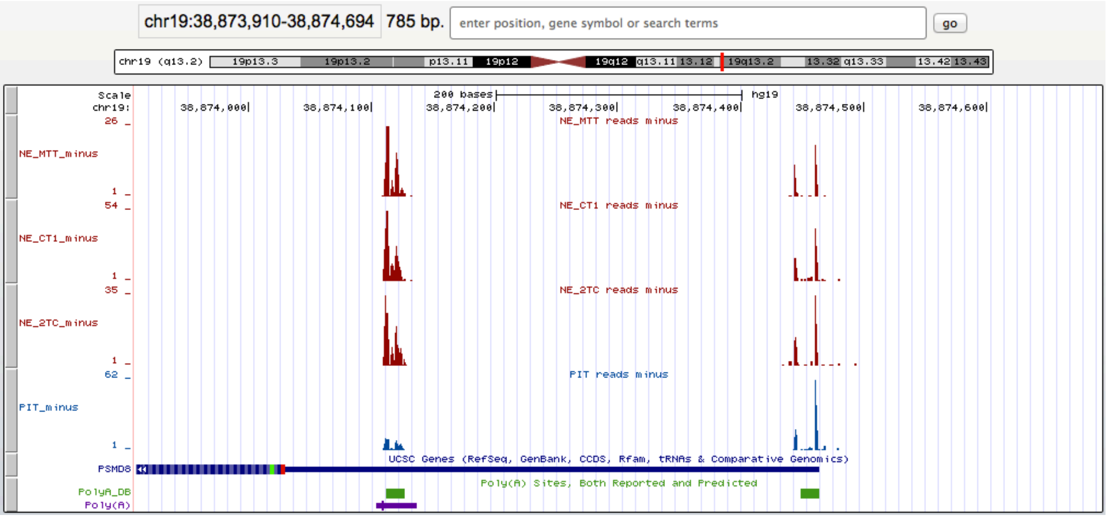

PSMD8

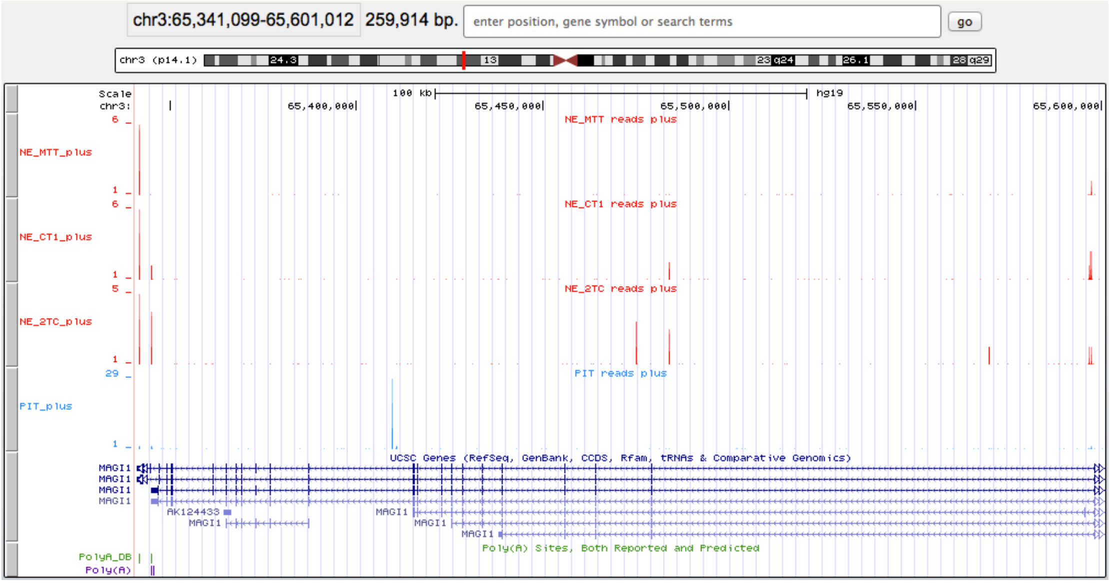

MAGI1

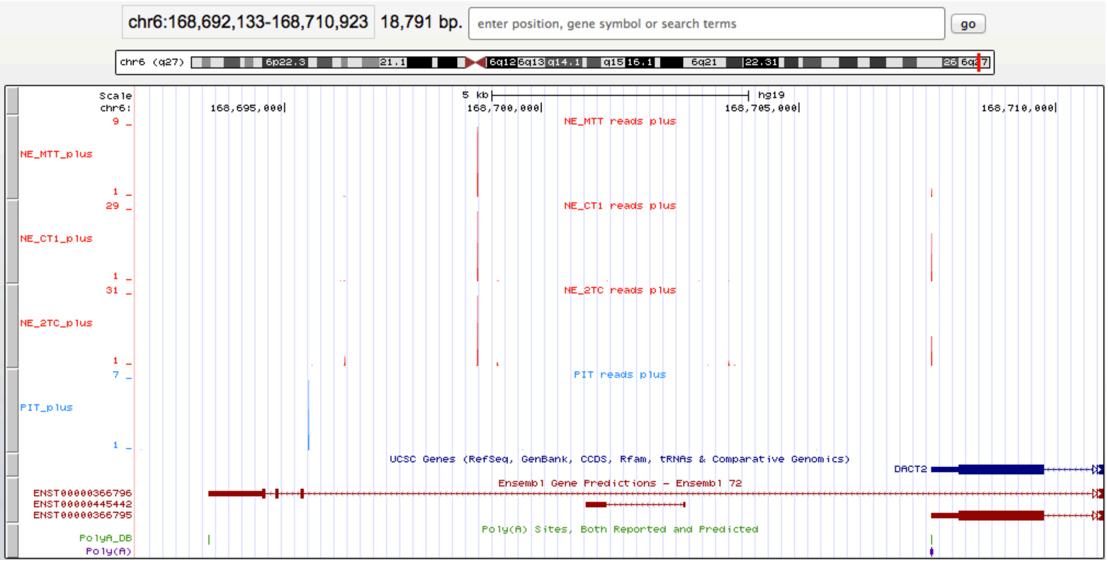

DACT2

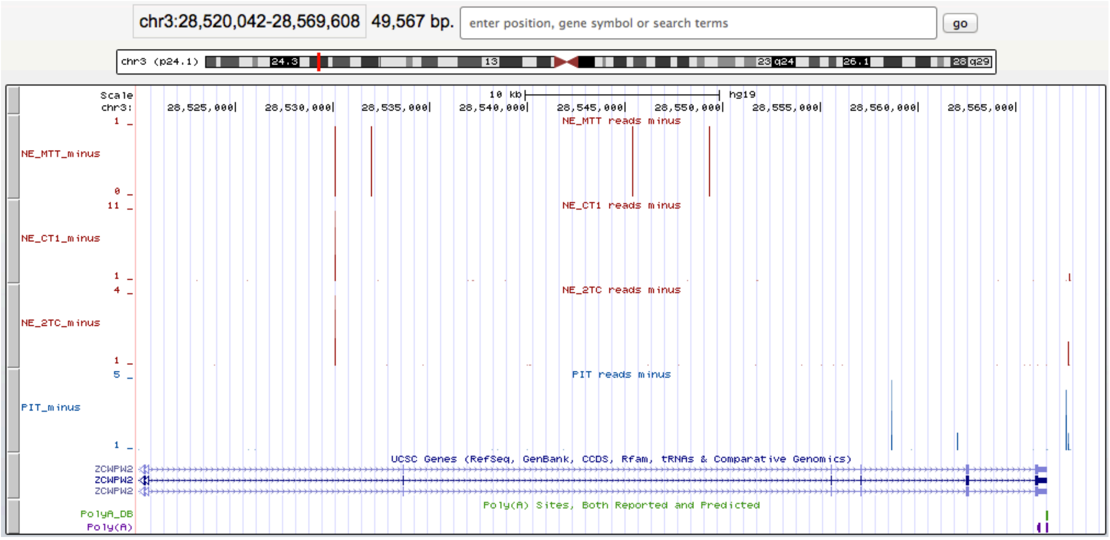

ZCWPW2

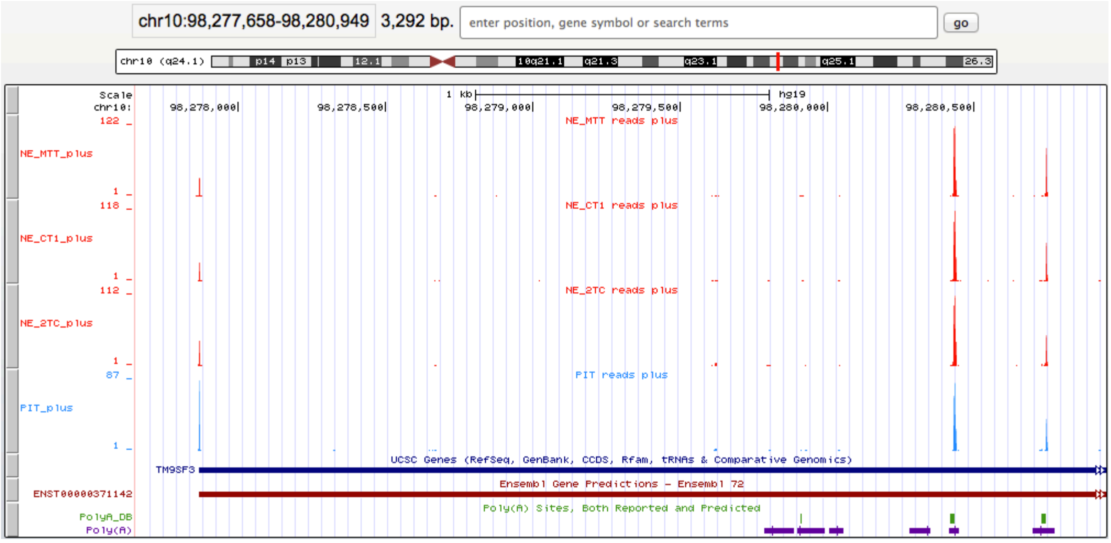

TM9SF3

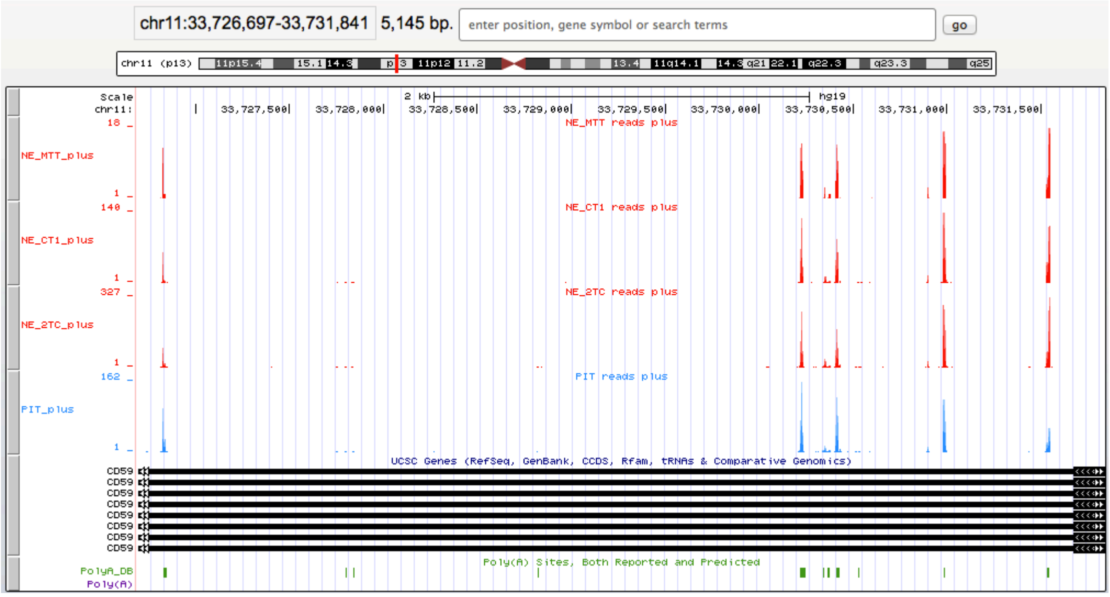

CD59

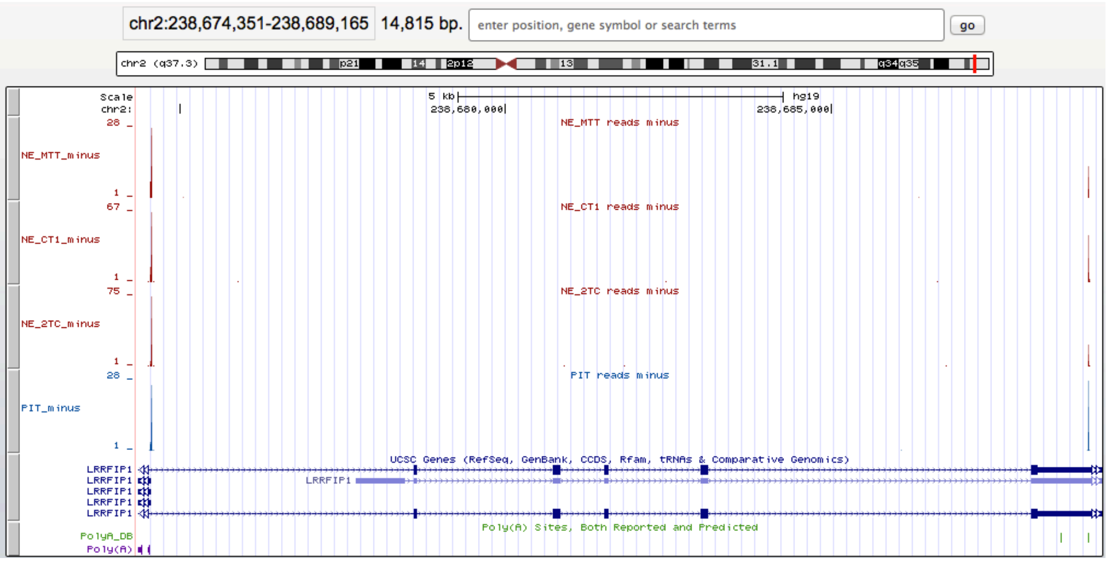

LRRFIP1

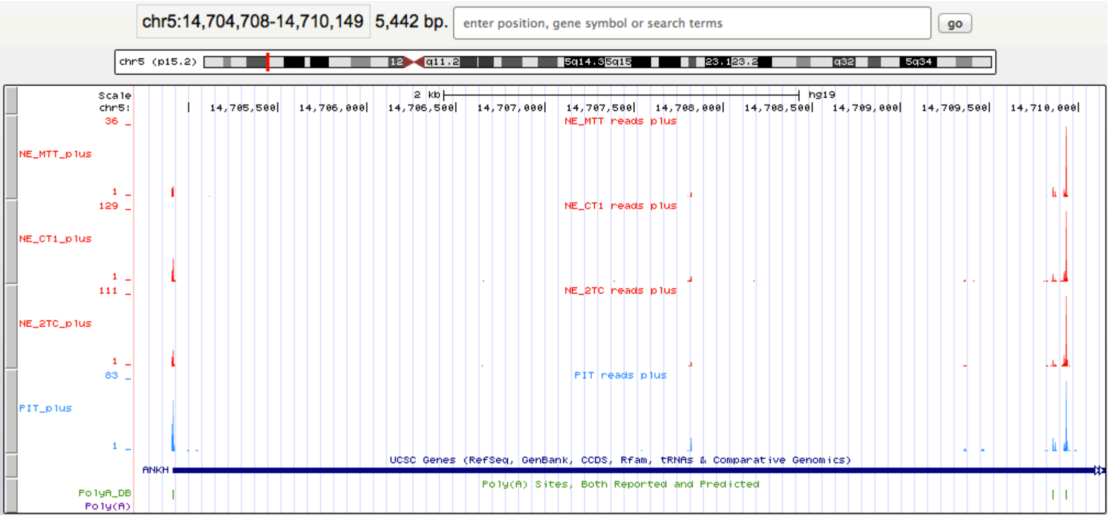

ANKH

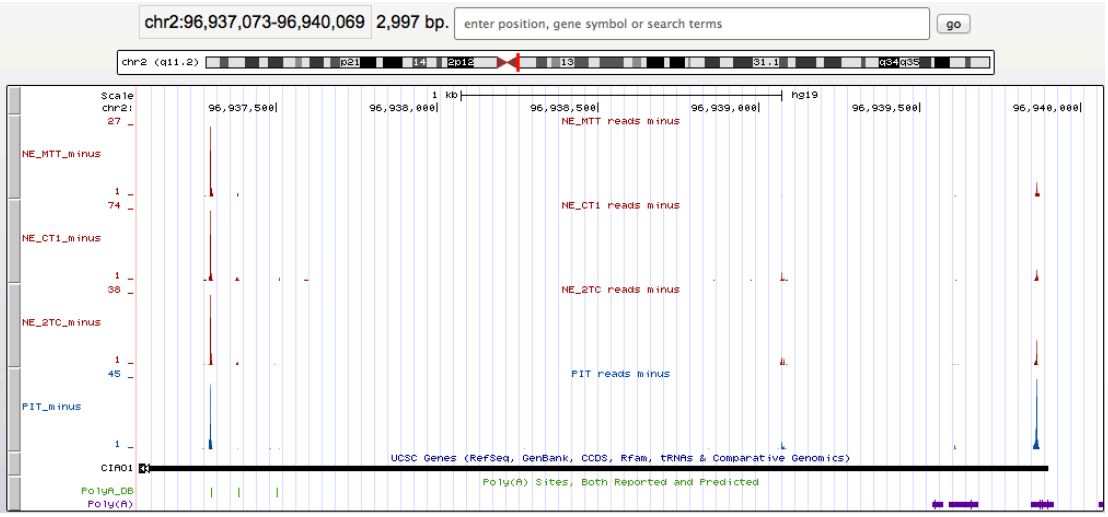

CIAO1

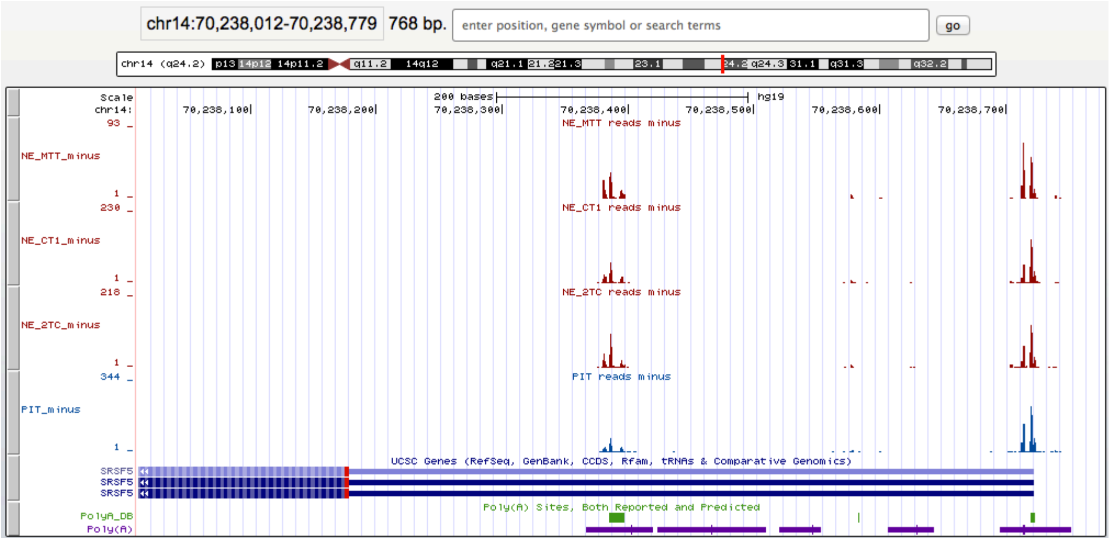

SRSF5

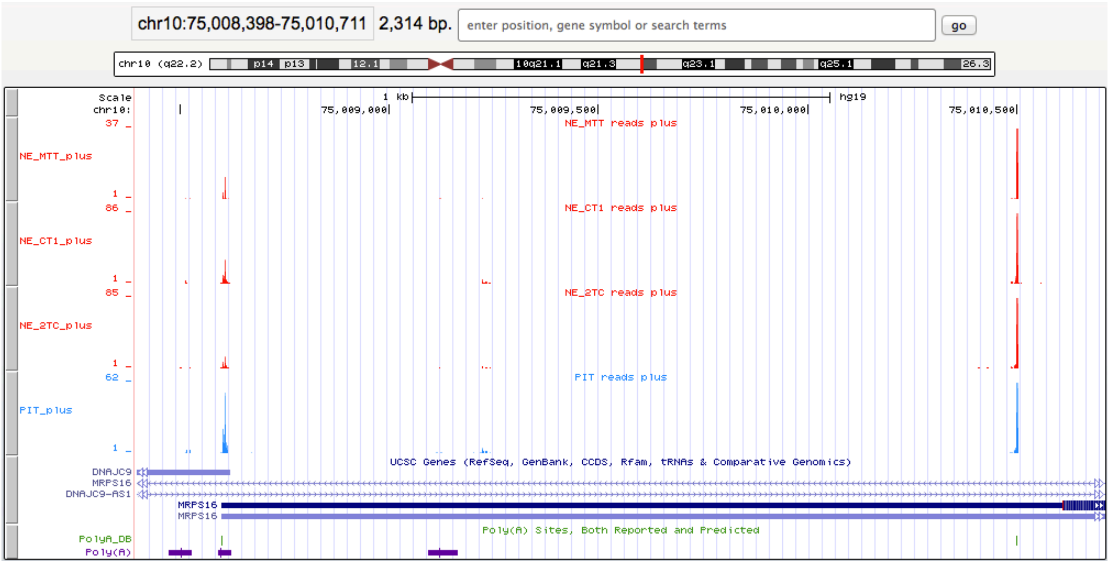

MRSP16

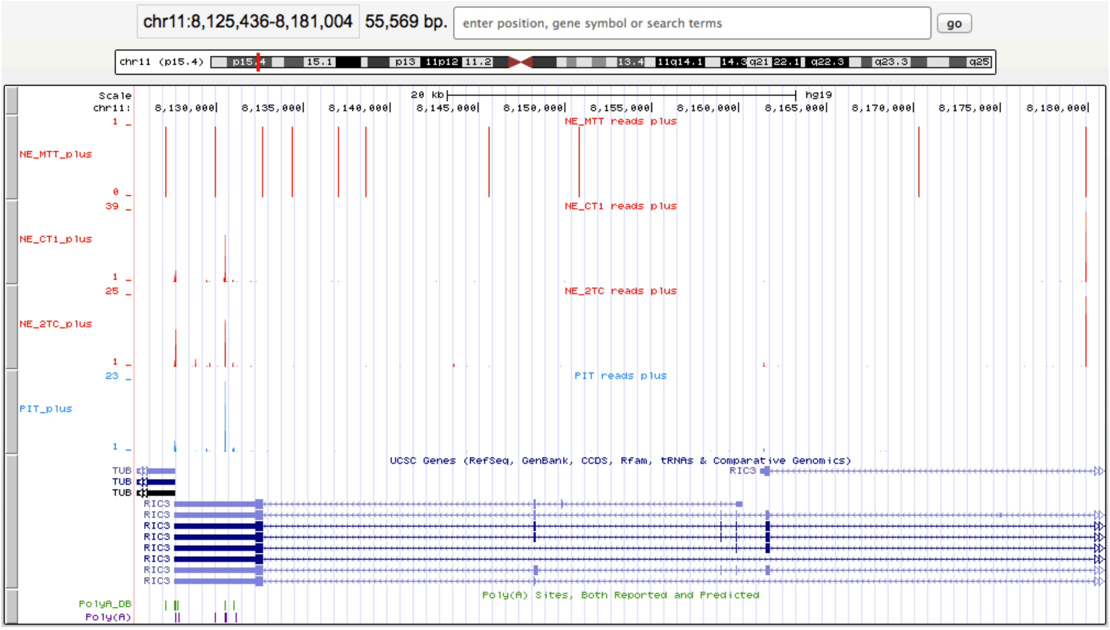
chr11:8,125,436-8,181,004
55,569 bp.
enter position, gene symbol or search terms
go
chr11 (p15.4)
Scale
chr11:
20 kb
hg19
8,130,000
8,135,000
8,140,000
8,145,000
8,150,000
8,155,000
8,160,000
8,165,000
8,170,000
8,175,000
8,180,000
NE_MTT reads plus
NE_MTT_plus
NE_CT1 reads plus
NE_CT1_plus
NE_2TC reads plus
NE_2TC_plus
PIT reads plus
PIT_plus
UCSC Genes (RefSeq, GenBank, CCDS, Rfam, tRNAs & Comparative Genomics)
TUB
RIC3
Poly(A) Sites, Both Reported and Predicted
PolyA_DB
Poly(A)

RIC3

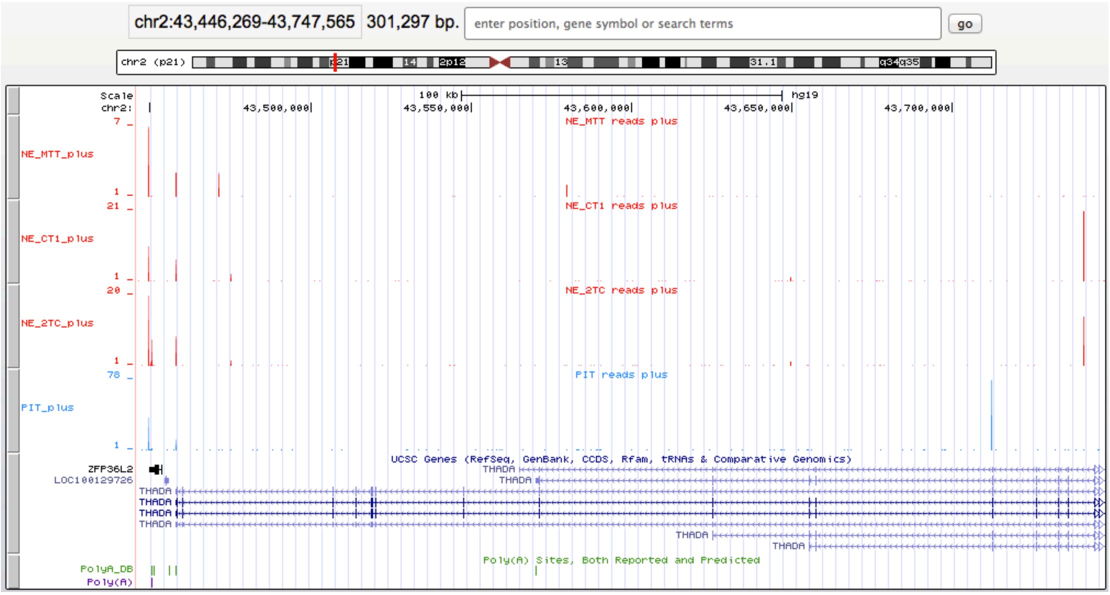
chr2:43,446,269-43,747,565
301,297 bp.
enter position, gene symbol or search terms
go
chr2 (p21)
Scale
chr2:
100 kb
hg19
43,500,000
43,550,000
43,600,000
43,650,000
43,700,000
NE_MTT reads plus
NE_MTT_plus
NE_CT1 reads plus
NE_CT1_plus
NE_2TC reads plus
NE_2TC_plus
PIT reads plus
PIT_plus
UCSC Genes (RefSeq, GenBank, CCDS, Rfam, tRNAs & Comparative Genomics)
ZFP36L2
LOC100129726
THADA
Poly(A) Sites, Both Reported and Predicted
PolyA_DB
Poly(A)

THADA

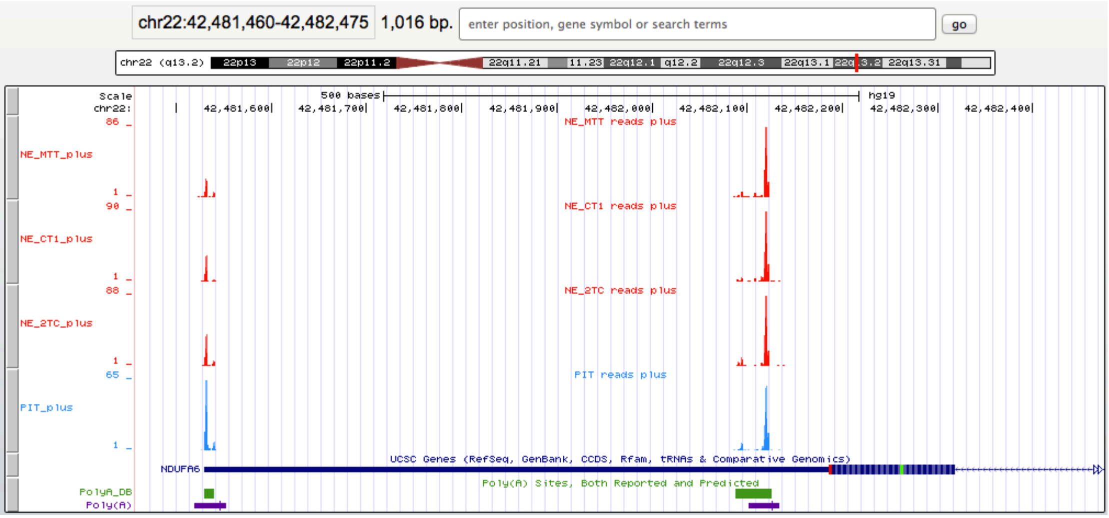
chr22:42,481,460-42,482,475
1,016 bp.
enter position, gene symbol or search terms
go
Scale
500 bases
hg19
chr22:
42,481,600
42,481,700
42,481,800
42,481,900
42,482,000
42,482,100
42,482,200
42,482,300
42,482,400
NE_MTT reads plus
NE_MTT_plus
NE_CT1 reads plus
NE_CT1_plus
NE_2TC reads plus
NE_2TC_plus
PIT reads plus
PIT_plus
UCSC Genes (RefSeq, GenBank, CCDS, Rfam, tRNAs & Comparative Genomics)
NDUFA6
Poly(A) Sites, Both Reported and Predicted
PolyA_DB
Poly(A)

NDUFA6
