# Supplementary material for: Alternative Polyadenylation of Tumor Suppressor Genes in Small Intestinal Neuroendocrine Tumors
Source: Front Endocrinol (Lausanne). 2014 Apr 15;5:46. doi: 10.3389/fendo.2014.00046 (PMC3995063; doi:10.3389/fendo.2014.00046)
Supplement: Supplementary file 2 [file Data_Sheet2.PDF]

Supplementary figure 2: Images of miRNA target sites in the 3’ UTRs of the 9 genes with UTR-APA, generated using the UCSC Genome Browser. The three tracks in red: NE_MTT_minus/plus, NE-CT1_minus/plus and NE_2TC_minus/plus represent the three small intestinal tumor samples. The track in blue: PIT_minus/plus represents the reference pituitary sample. miRNA target sites are shown above the tracks in black.

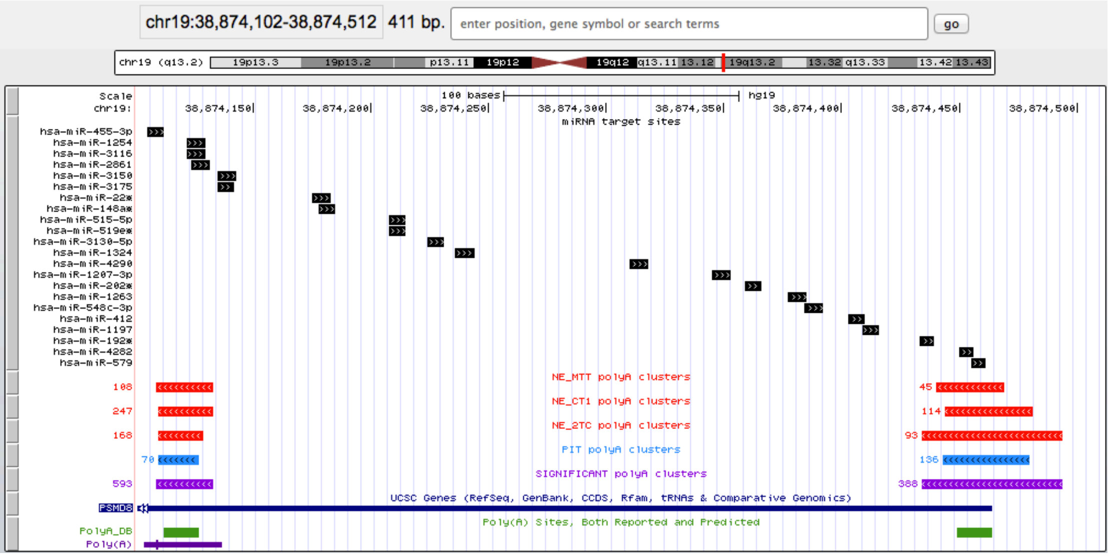

PSMD8

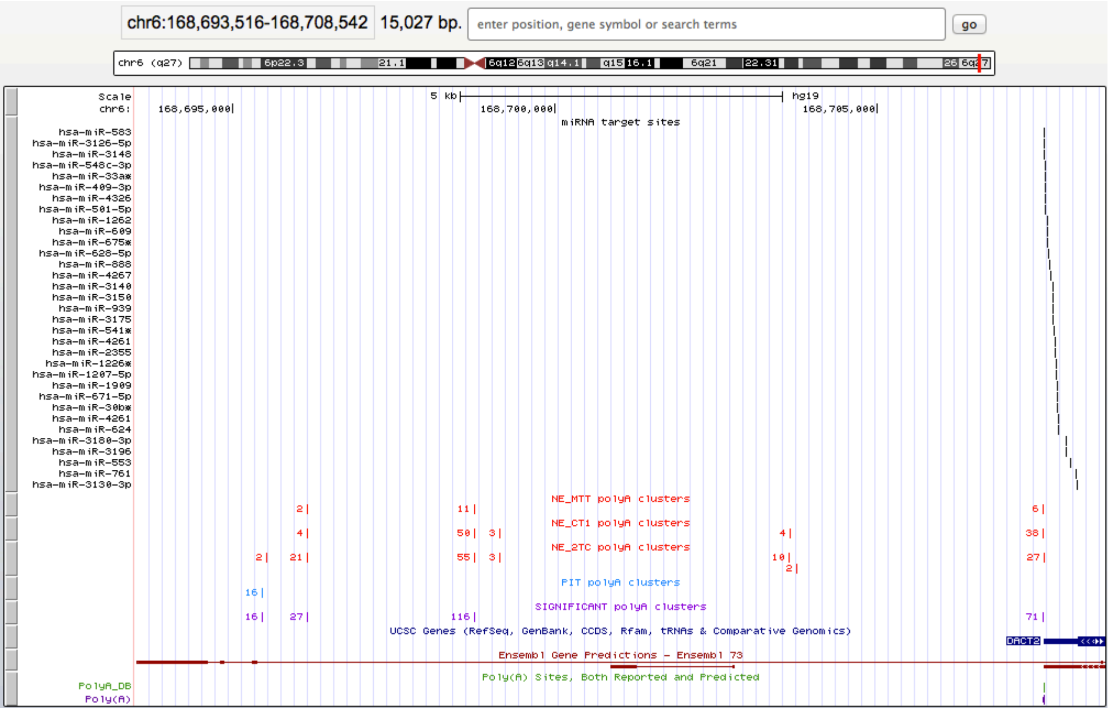

DACT2

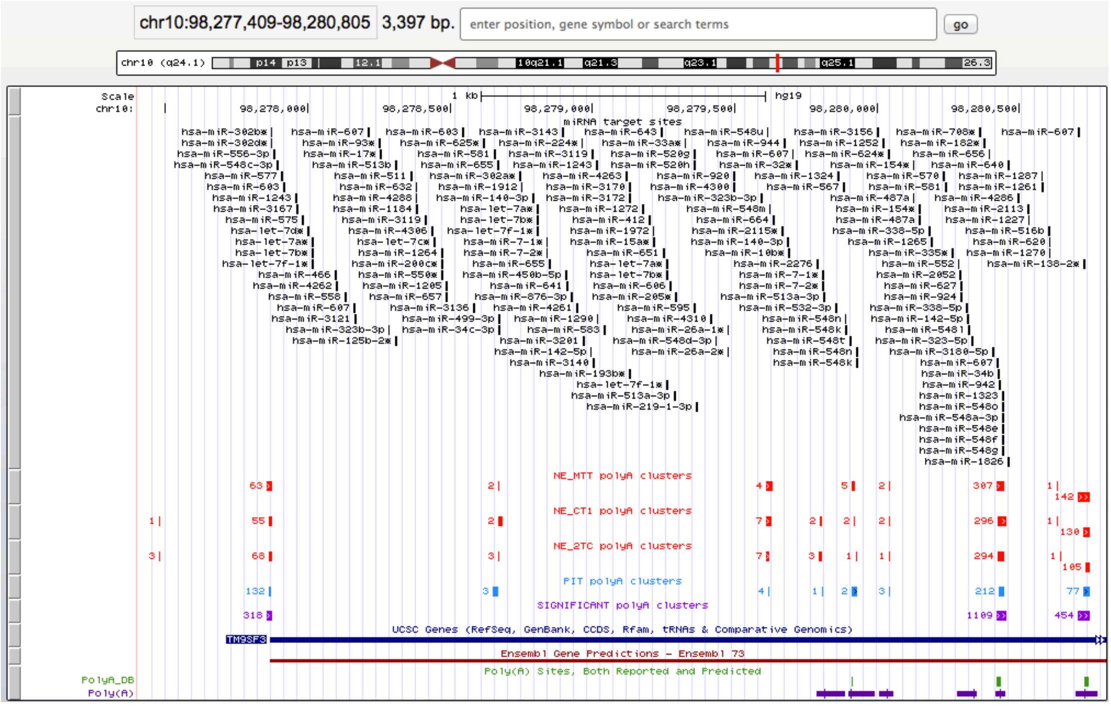
chr10:98,277,409-98,280,805 3,397 bp.
enter position, gene symbol or search terms
go
miRNA target sites
NE_MTT polyA clusters
NE_CT1 polyA clusters
NE_2TC polyA clusters
PIT polyA clusters
SIGNIFICANT polyA clusters
UCSC Genes (RefSeq, GenBank, CCDS, Rfam, tRNAs & Comparative Genomics)
TM9SF3
Ensembl Gene Predictions - Ensembl 73
Poly(A) Sites, Both Reported and Predicted
PolyA_DB
Poly(A)

TM9SF3

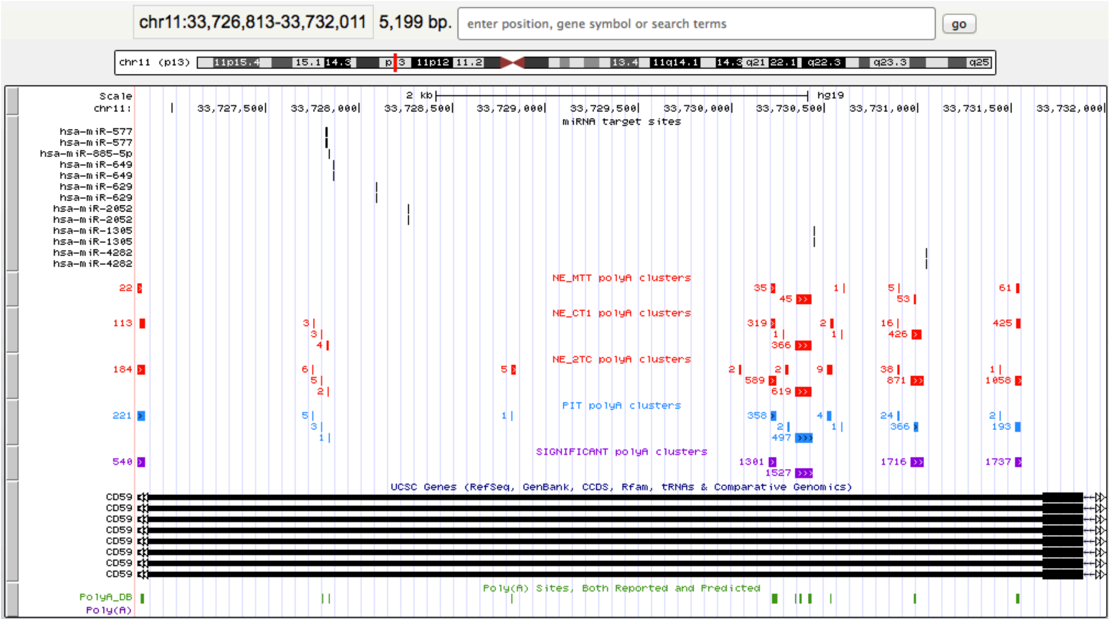
chr11:33,726,813-33,732,011 5,199 bp.
enter position, gene symbol or search terms
go
miRNA target sites
NE_MTT polyA clusters
NE_CT1 polyA clusters
NE_2TC polyA clusters
PIT polyA clusters
SIGNIFICANT polyA clusters
UCSC Genes (RefSeq, GenBank, CCDS, Rfam, tRNAs & Comparative Genomics)
CD59
Poly(A) Sites, Both Reported and Predicted
PolyA_DB
Poly(A)

CD59

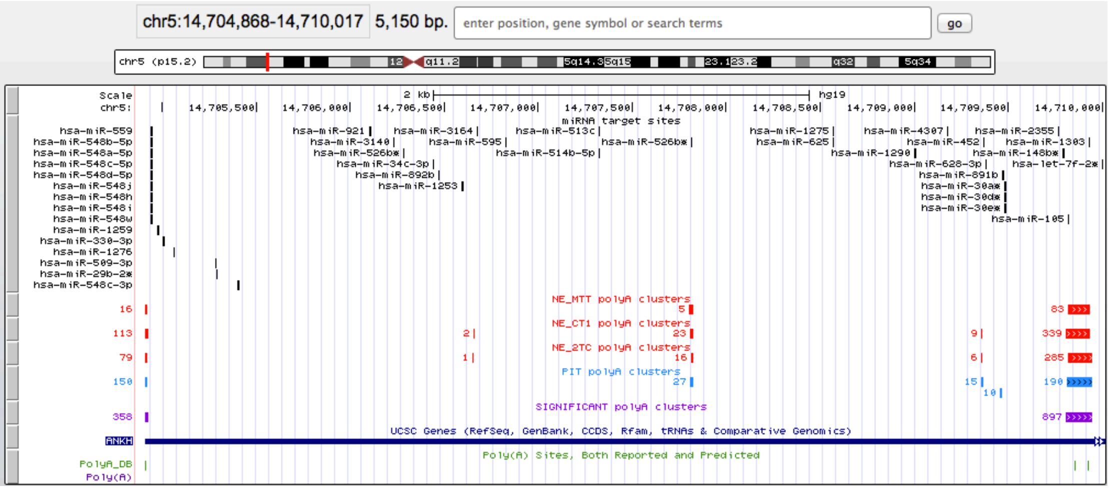
chr5:14,704,868-14,710,017 5,150 bp.
enter position, gene symbol or search terms
go
miRNA target sites
NE_MTT polyA clusters
NE_CT1 polyA clusters
NE_2TC polyA clusters
PIT polyA clusters
SIGNIFICANT polyA clusters
UCSC Genes (RefSeq, GenBank, CCDS, Rfam, tRNAs & Comparative Genomics)
ANKH
Poly(A) Sites, Both Reported and Predicted
PolyA_DB
Poly(A)

ANKH

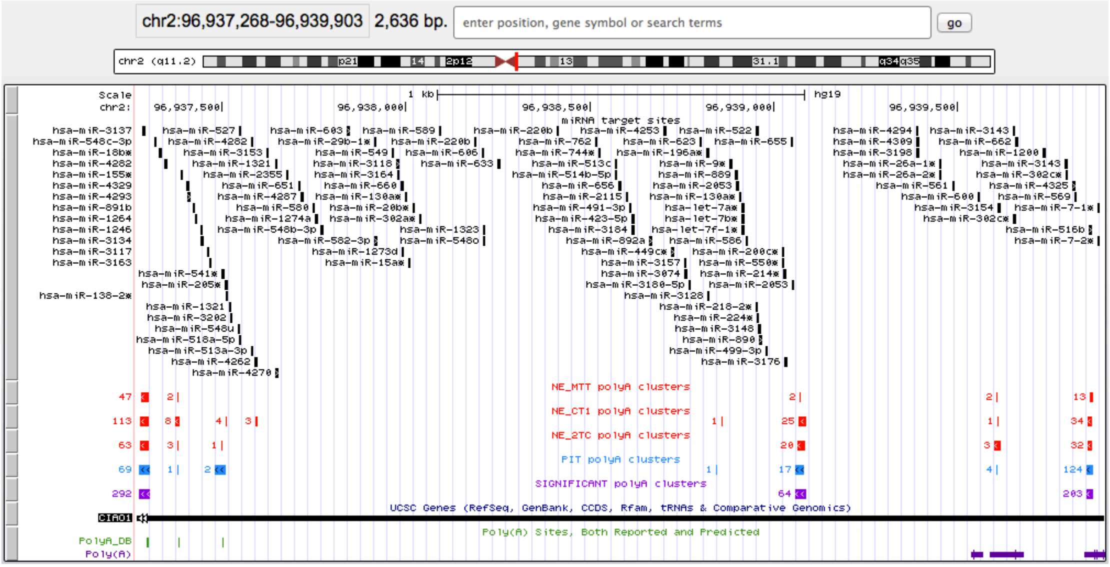
chr2:96,937,268-96,939,903 2,636 bp.
enter position, gene symbol or search terms
go
miRNA target sites
NE_MTT polyA clusters
NE_CT1 polyA clusters
NE_2TC polyA clusters
PIT polyA clusters
SIGNIFICANT polyA clusters
UCSC Genes (RefSeq, GenBank, CCDS, Rfam, tRNAs & Comparative Genomics)
CIAO1
Poly(A) Sites, Both Reported and Predicted
PolyA_DB
Poly(A)

CIAO1

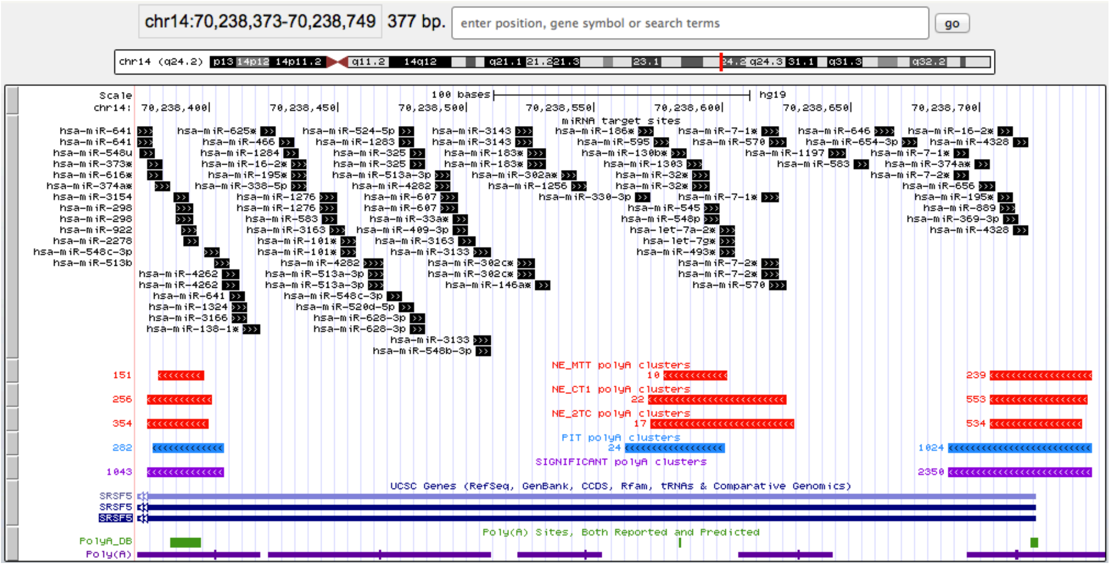

SRSF5

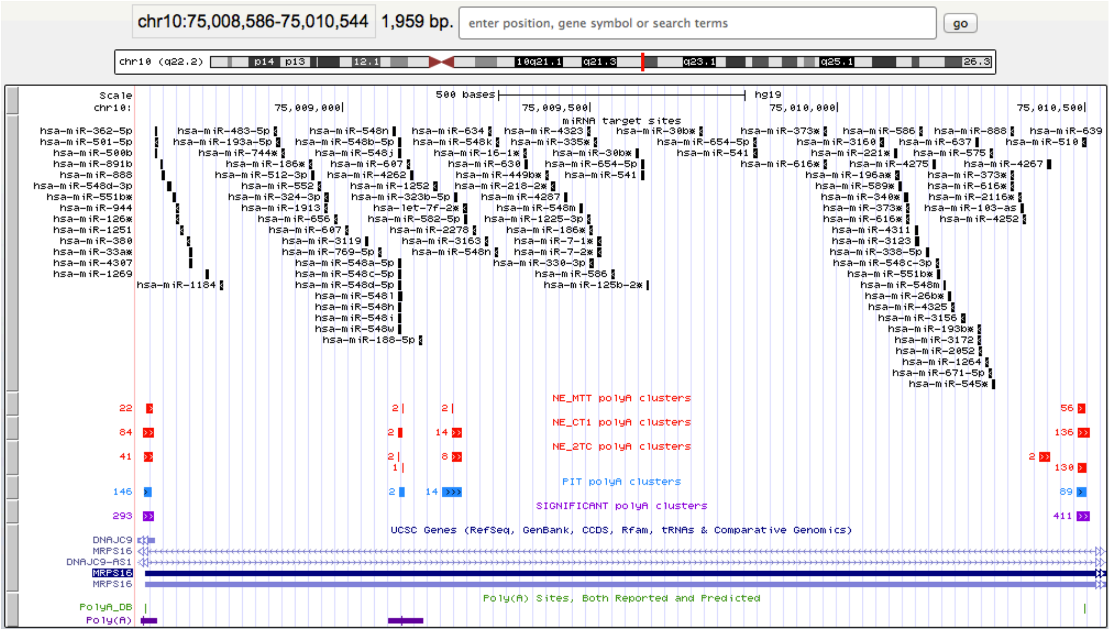

MRSP16

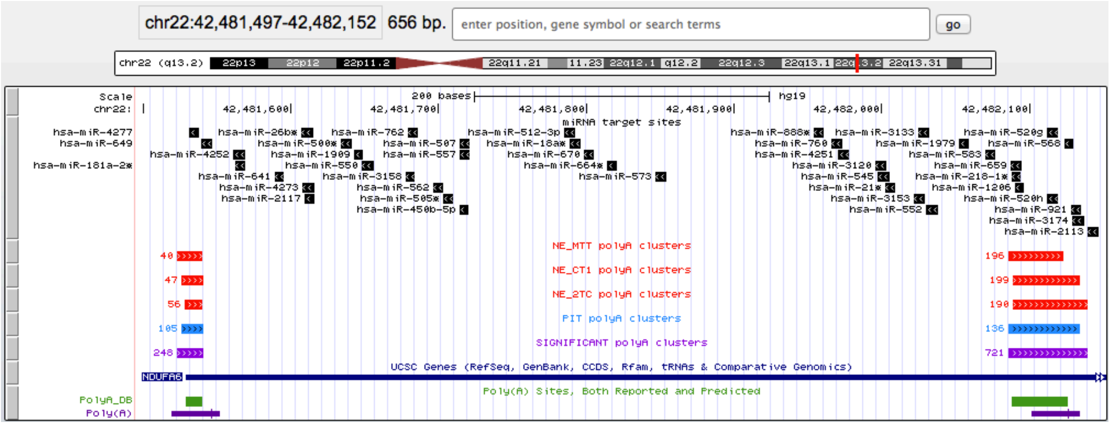
chr22:42,481,497-42,482,152 656 bp.
enter position, gene symbol or search terms
go
miRNA target sites
NE_MTT polyA clusters
NE_CT1 polyA clusters
NE_2TC polyA clusters
PIT polyA clusters
SIGNIFICANT polyA clusters
UCSC Genes (RefSeq, GenBank, CCDS, Rfam, tRNAs & Comparative Genomics)
NDUFA6
Poly(A) Sites, Both Reported and Predicted
PolyA_DB
Poly(A)

NDUFA6
